# Differences in the Effect of Internet-Based Cognitive Behavioral Therapy for Improving Nonclinical Depressive Symptoms Among Workers by Time Preference: Randomized Controlled Trial

**DOI:** 10.2196/10231

**Published:** 2018-08-10

**Authors:** Kotaro Imamura, Toshi A Furukawa, Yutaka Matsuyama, Akihito Shimazu, Kazuto Kuribayashi, Kiyoto Kasai, Norito Kawakami

**Affiliations:** ^1^ Department of Mental Health Graduate School of Medicine The University of Tokyo Tokyo Japan; ^2^ Departments of Health Promotion and Human Behavior and of Clinical Epidemiology Kyoto University Graduate School of Medicine/School of Public Health Kyoto University Kyoto Japan; ^3^ Department of Biostatistics Graduate School of Medicine The University of Tokyo Tokyo Japan; ^4^ Center for Human and Social Sciences Kitasato University College of Liberal Arts and Sciences Sagamihara Japan; ^5^ Department of Psychiatric Nursing Graduate School of Medicine The University of Tokyo Tokyo Japan; ^6^ Department of Neuropsychiatry Graduate School of Medicine The University of Tokyo Tokyo Japan

**Keywords:** internet-based computerized cognitive behavioral therapy, time preference, nonclinical depressive symptoms, workers

## Abstract

**Background:**

Previous randomized controlled trials (RCTs) have shown a significant intervention effect of internet-based computerized cognitive behavioral therapy (iCBT) on improving nonclinical depressive symptoms among healthy workers and community residents in a primary prevention setting. Time preference is one’s relative valuation for having a reward (eg, money) at present than at a later date. Time preference may affect the effectiveness of cognitive behavioral therapy.

**Objective:**

This RCT aimed to test the difference of intervention effect of an iCBT program on improving nonclinical depressive symptoms between two subgroups classified post-hoc on the basis of time preference among workers in Japan.

**Methods:**

All workers in one corporate group (approximate n=20,000) were recruited. Participants who fulfilled the inclusion criteria were randomly allocated to either intervention or control groups. Participants in the intervention group completed 6 weekly lessons and homework assignments within the iCBT program. The Beck Depression Inventory-II (BDI-II) and Kessler’s Psychological Distress Scale (K6) measures were obtained at baseline and 3-, 6-, and 12-month follow-ups. Two subgroups were defined by the median of time preference score at baseline.

**Results:**

Only few (835/20,000, 4.2%) workers completed the baseline survey. Of the 835 participants, 706 who fulfilled the inclusion criteria were randomly allocated to the intervention or control group. Participants who selected irrational time preference options were excluded (21 and 18 participants in the intervention and control groups, respectively). A three-way interaction (group [intervention/control] × time [baseline/follow-up] × time preference [higher/lower]) effect of iCBT was significant for BDI-II (*t*_1147.42_=2.33, *P*=.02) and K6 (*t*_1254.04_=2.51, *P*=.01) at the 3-month follow-up, with a greater effect of the iCBT in the group with higher time preference. No significant three-way interaction was found at the 6- and 12-month follow-ups.

**Conclusions:**

The effects of the iCBT were greater for the group with higher time preference at the shorter follow-up, but it was leveled off later. Workers with higher time preference may change their cognition or behavior more quickly, but these changes may not persist.

**Trial Registration:**

UMIN Clinical Trials Registry UMIN000014146; https://upload.umin.ac.jp/cgi-open-bin/ctr_e/ctr_view.cgi? recptno=R000016466 (Archived by WebCite at http://www.webcitation.org/70o2rNk2V)

## Introduction

Depressive disorder is one of the most prevalent psychiatric disorders, affecting around 340 million people worldwide [1] and is associated with a substantial deterioration in quality of life and economic loss in the community and the workplace [2,3]. The primary prevention of depressive disorder is an important strategy for global mental health. The presence of nonclinical depressive symptoms (ie, subthreshold depressive symptoms) is associated with high prospective risk of developing major depressive disorder (MDD) [4,5], and a previous meta-analysis reported that it was possible to prevent the onset of MDD using psychological interventions by targeting individuals with no diagnosed depression at baseline survey [6].

One of the most effective psychological interventions for depression is cognitive behavioral therapy (CBT) [7], and internet-based computerized CBT (iCBT) has received attention in recent years because it is less expensive, more easily administered, and potentially more accessible than conventional CBT. Previous randomized controlled trials (RCTs) have shown a significant intervention effect of iCBT for improving nonclinical depressive symptoms [8,9] and preventing the onset of new major depressive episodes (MDEs) [10] among healthy workers and community residents.

Recently, variables that might predict treatment response to CBT for depression have been investigated. Previous studies have reported that the severity of depressive symptoms at baseline and the rate of change in depressive symptom severity within 5 treatment sessions significantly predicted treatment response to CBT [11,12]. As a predictor of treatment response to iCBT, there was a significant association with pretreatment severity of depression, gender, marital status, and education [13-15]. In addition, a recent study reported that individual differences in reward processing, measured by reward positivity, contribute to the effectiveness of CBT for depression [16]. This result implies that CBT may decrease depressive symptoms by enhancing the brain’s reward function. Sensitivity for reward may be an important predictor of the effectiveness of CBT.

Time preference (or time discounting) has attracted interest in the field of behavioral economics and behavioral medicine as a potentially common factor of multiple behaviors that pose risks for health [17-19]. Time preference is one’s relative reward valuation (eg, money) at present than at a later date [20]. Frederick et al (2002) stated that time discounting means caring less about a future consequence, including factors that diminish the expected utility generated by a future consequence, and time preference refers to the preference for immediate over delayed utility [20]. Time preference can be shortly defined as the degree to which people prefer present to future satisfaction [21]. Individuals that have a high rate of time preference or tend to prefer utility in the present are often designated as present-oriented and labeled as impatient. On the other hand, individuals with a low rate of time preference or those who tend to prefer future utilities are often designated as future-oriented and are said to be patient [22]. Time preference may affect the effectiveness of CBT for various reasons. First, people with higher time preference may be less eager to participate in a health education program for preventing future psychological distress or mental disorders, such as CBT. Second, people with higher time preference may be less willing to change their behaviors in order to improve their future health [17-19]. Third, for the same reason, the effect of CBT may not be persistent among people with higher time preference. However, no previous study has investigated the effect of time preference on the intervention effect of CBT for improving nonclinical depression. Investigating the impact of time preference on the effectiveness of CBT would contribute to the development of a theory for behavioral determinants of the effectiveness of CBT. In practice, it would also lead to identifying a subgroup for which CBT is less effective and allow us to improve interventions and treatment effects for this population.

This RCT aimed to examine whether an iCBT program was effective in improving nonclinical depressive symptoms among healthy workers in Japan, at 3-, 6-, and 12-month follow-ups, particularly to test the difference of the intervention effect between two subgroups classified post-hoc on the basis of time preference: a lower time preference subgroup and a higher time preference subgroup.

## Methods

### Trial Design

This study was a randomized controlled trial. The allocation ratio of the intervention group to the control group was 1:1. The Research Ethics Review Board of the Graduate School of Medicine and Faculty of Medicine, the University of Tokyo approved the study procedures (no. 3083-2). The study protocol was registered at the University Hospital Medical Information Network Clinical Trials Registry (UMIN000014146). The protocol article for this trial is available [23]. This study focused on the first-year recruitment for the planned larger study. The original protocol of this RCT aims to investigate whether an iCBT program could prevent the onset of MDE as a primary outcome. Outcomes in this study (ie, depressive symptoms and psychological distress) were collected as secondary outcomes. This manuscript was reported according to the Consolidated Standards of Reporting Trials guidelines.

### Participants

All workers in one corporate group (the total employee population, approximately 20,000) were recruited from one of the major telecom carrier companies in Japan by an invitation email from their internal employee assistance program staff in March 2015. Those who were interested in participating in the study were asked to go to a research website to obtain a full explanation of the study’s aim. Consent from a respondent was obtained when he or she completed a baseline questionnaire. Before the Web-based baseline survey, participants were invited to read the explanation on the research website and asked to click on an “agree” button to show their consent to participate in the study; then they proceeded to the baseline questionnaire page. Written consent was not required by the National Ethical Guidelines for Epidemiologic Research, Japan; the Research Ethics Review Board of Graduate School of Medicine and Faculty of Medicine, the University of Tokyo, approved this procedure for obtaining participants’ consents.

The inclusion criteria at the baseline survey were as follows: (1) age 20-60 years at the study entry, (2) currently employed full-time by the company, and (3) being able to access the internet via a PC at home or at their workplace. The exclusion criteria were as follows: (1) nonregular or part-time employees, (2) having an MDE in the past month, based on the diagnostic criteria on the web version of World Health Organization Composite International Diagnostic Interview 3.0 [24], (3) having lifetime history of bipolar disorder (World Health Organization Composite International Diagnostic Interview 3.0), (4) on sick leave for 15 or more days for a physical or mental condition in the past 3 months, and (5) undergoing current treatment for a mental health problem.

### Intervention

Participants assigned to an intervention group participated in the iCBT program called *the Internet CBT program; useful mental health solutions series for business*. Please refer for the details of this program elsewhere [23]. Briefly, the program was a 6-week, 6-lesson, Web-based training course to provide CBT-based stress management skills via one 30-minute lesson per week. The CBT components of the program included self-case formulation, cognitive restructuring, assertiveness, problem-solving, and relaxation. At the end of each lesson, the participants were asked to submit homework to facilitate their understanding, but on voluntary basis. Participants who submitted their homework received feedback from trained clinical psychologists.

### Intervention Group

Participants in the intervention group completed 6 weekly lessons and homework within the iCBT program. They were allowed to complete the 6 lessons and submit their homework within 10 weeks after the baseline survey. The participants were reminded by email to complete each lesson and to submit their homework if they had not already done so. Reminders were sent from the research office to the participants every Monday.

### Control Group

Participants in the control group were able to use an internal employee assistance program service, such as consulting with a physician or a psychologist, and group or Web-based education/training programs for promoting mental health as a treatment as usual. These programs contained few descriptions of CBT knowledge and skills.

### Outcome

All outcomes were measured using a Web-based self-report questionnaire at baseline and 3-, 6-, and 12-month follow-ups.

#### Depressive Symptoms

The Beck Depression Inventory-II (BDI-II) is a 21-item self-report inventory that measures depressive symptoms such as sadness, pessimism, suicidal thoughts or wishes, tiredness or fatigue, loss of energy, and loss of pleasure, among others [25,26]. Each item was scored on a scale ranging from 0 to 3, with a higher score indicating more serious depressive symptoms.

#### Psychological Distress

Kessler’s Psychological Distress Scale (K6) consists of 6 items assessing the frequency with which respondents experienced symptoms of psychological distress during the past 30 days [27,28]. The response options range from 0 (*none of the time*) to 4 (*all of the time*). The internal reliability and validity found in previous studies were acceptable [27].

#### Time Preference

In this study, time preference was assessed by the following procedure [29,30]. The respondents were asked to choose between two options, A or B. The respondent would receive 1 million yen (approximately US $12,000) in 1 month upon choosing option A, or a different amount to be received in 13 months upon choosing option B. This question comprised 9 choices with each annual interest rate ranging from −5% to ≥10% (Multimedia Appendix 1). For instance, individuals who tended to choose option B, despite lower annual interest in 13 months, were considered more future-oriented (ie, lower time preference). On the other hand, individuals who tended to choose option A, despite higher annual interest in 13 months, were considered more present-oriented (ie, higher time preference).

In this study, we defined two subgroups according to the median time preference score at baseline because the concept of time preference has no clear cutoff point. One was the *lower time preference* subgroup (ie, the participants who had low levels of time preference and selected the 0.1%-6% annual interest rate), and the other was the *higher time preference* subgroup (ie, the participants who had high levels of time preference and selected the 10% annual interest rate or more). Participants who selected irrational options (interest rate, −5% or 0%) were excluded.

#### Demographic Characteristics

Demographic data such as age, gender, marital status, occupation, education, and chronic disease were also collected.

### Sample Size

We determined that to detect an effect size, a minimum sample size of 4136 in each group was necessary. This calculation considered an incidence ratio of 0.62 or greater for the onset of an MDE, at an alpha error rate of 0.05 (two-tailed) and a beta error rate of 0.20, with an expected dropout rate of 25%.

No previous study reported an effect size for a difference of intervention effect between lower and higher time preference groups. The estimated post-hoc power (1-beta) was 0.54 if the effect size was 0.2, assuming that the alpha was less than 0.05 (two-tailed), and 70% (314/448) of the initial 448 respondents in the lower time preference subgroup and 219 respondents in the higher time preference subgroup respondents completed the follow-up using the G*Power 3 program [31,32].

### Randomization

Participants who fulfilled the inclusion criteria were randomly assigned to an intervention or control group. Stratified permuted-block randomization was conducted as well. Participants were stratified into two strata according to K6 score (5 or greater or less than 5) on the baseline survey. A stratified permuted-block random table was generated by an independent biostatistician. Enrollment was conducted by a clinical research coordinator, and assignment was conducted by an independent research assistant. The stratified permuted-block random table was password protected and kept blind to the researcher. Only the research assistant was able to access it for random allocation. A prestratification for randomization by time preference was not conducted.

### Statistical Methods

Primary analyses were conducted for the whole sample. For main analysis, a mixed model for repeated measures conditional growth model analysis was conducted to estimate the fixed effect of a three-way interaction as an indicator of intervention effect: group (intervention and control) × time (baseline and 1-, 6-, and 12-month follow-ups) × subgroup (lower time preference and higher time preference). For sensitivity analysis, a mixed model for repeated measures analysis of variance was conducted to estimate the fixed effect of three-way interaction as an indicator of intervention effect at each follow-up: group (intervention and control) × time (baseline and 1-, 6-, or 12-month follow-up) × subgroup (lower time preference and higher time preference). In these analyses, two models were applied. Model 1 was crude (not adjusted). Model 2 was adjusted by the potential confounders: gender, education, and occupation. All analyses were conducted according to the intention-to-treat principles. The MIXED procedure in SPSS Statistics 21.0 (IBM Corp, Armonk, NY, USA) was used.

Secondary analyses were conducted for all respondents as well as separately for each subgroup. A mixed model for repeated measures conditional growth model analysis was conducted to estimate the fixed effect of a group (intervention and control) × time (baseline, 1-, 6-, and 12-month follow-ups) interaction as an indicator of intervention effect. As a sensitivity analysis, a mixed model for repeated measures analysis of variance was conducted to estimate the fixed effect of a group (intervention and control) × time (baseline and 1-, 6-, or 12-month follow-up) interaction as an indicator of the intervention effect at each follow-up.

In addition, the effect sizes were calculated using estimated means based on the MIXED procedure among all respondents in each subgroup. First, estimated mean differences between baseline and follow-ups of each intervention and control group were calculated. Next, the effect sizes (ESs) were calculated by dividing between differences of the intervention and control groups by pooled SDs, which were calculated using respondents who completed the questionnaire at baseline and at follow-ups. The values of 0.2, 0.5, and 0.8 were interpreted as small, medium, and large ESs, respectively [33].

As a process evaluation, the rate (percentage) of completers of lessons and submitters of iCBT program homework were calculated among participants in the intervention group, for each lower and higher time preference subgroup.

## Results

### Recruitment

Recruitment and the baseline survey were conducted in March 2015. The intervention and control groups were assessed at approximately 3 months (June 2015), 6 months (September 2015), and 12 months (March 2016) after the baseline survey.

**Figure 1:**
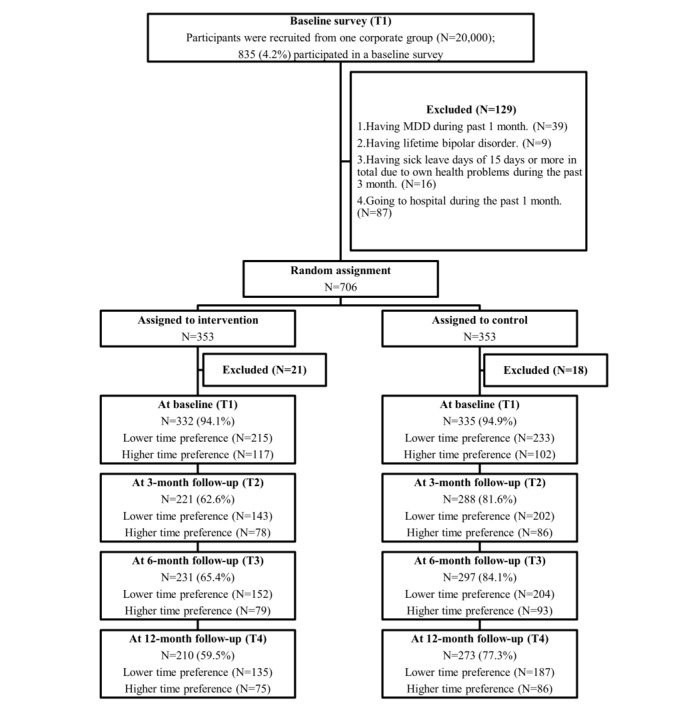
Participant flowchart. MDD: major depressive disorder.

The participant flowchart is shown in Figure 1. In total, 4.2% of workers (835/20,000) participated in a baseline survey. Out of those workers, 706 met the eligibility criteria of this study and 129 were excluded (39 cases fulfilled exclusion criteria 1; 9 cases fulfilled exclusion criteria 2; 16 cases fulfilled exclusion criteria 3; 87 cases fulfilled exclusion criteria 4). Out of those excluded workers, a total of 13 cases fulfilled exclusion criteria 1 and 4; 2 cases fulfilled exclusion criteria 2 and 4; 5 cases fulfilled exclusion criteria 3 and 4; and 1 case fulfilled exclusion criteria 2, 3, and 4. Seven hundred and six participants were randomly allocated to the intervention or control group (n=353 for each). Figure 1 also shows excluded participants (21 in the intervention group and 18 in the control group) after randomization because they selected irrational time preference options (interest rate, −5% or 0%). At each follow-up, the response rate of the control group was higher than that of the intervention group. The reasons for dropping out were not assessed in this study.

### Baseline Characteristics

Demographic characteristics are presented in Table 1. Compared with the lower time preference subgroup, there were more males, managers, participants with a graduate school education, and those with chronic diseases in the higher time preference subgroup. In the whole sample, most participants were married, held clerical positions, received a university or higher education, and did not report having chronic diseases.

After excluding participants who selected irrational options (interest rate, −5% or 0%), we divided the total sample into two groups of participants: one with low levels of time preference (6% or lower annual interest rate) and one with high levels of time preference (10% or higher annual interest rate). The details of the number of respondents in each group are shown in Multimedia Appendix 1.

### Changing Outcomes by Groups During the Follow-Up

Tables 2 and 3 show the means and SDs of the outcome variables at baseline and 3-, 6-, and 12-month follow-ups in the intervention and control groups. In addition, we report estimated mean differences between the intervention and control groups, pooled SDs, and ESs in each lower and higher time preference subgroup. In the lower time preference subgroup, the estimated mean differences were −1.21 (ES=−0.30) on K6 at 12-month and −1.68 (ES=−0.23) and −2.63 (ES=−0.33) on BDI-II at 6- and 12-month follow-ups, respectively. In the higher time preference subgroup, the estimated mean differences were −1.13 (ES=−0.30) on K6 at 3-month follow-up and −2.55 (ES=−0.37) and −2.59 (ES=−0.41) on BDI-II at 3- and 6-month follow-ups, respectively.

### Interaction Effects of Internet-Based Computerized Cognitive Behavioral Therapy and Time Preference

Table 4 shows the estimated three-way interaction effects of iCBT on the outcome variables on the basis of the mixed model analyses. iCBT showed a significant effect on BDI-II and K6 at 3-month follow-up, and only a marginally significant effect on BDI-II at 6-month follow-up. These results were consistent with the results after adjusting for gender, occupational status, and education.

**Table 1:** Baseline characteristics of participants in the intervention and control groups, in each of the two subgroups.

Characteristic		Lower time preference^a^, mean (SD)		Higher time preference^b^, mean (SD)	
		Intervention (n=215)	Control (n=233)	Intervention (n=117)	Control (n=102)
Age (years)		38.7 (8.1)	39.0 (7.7)	39.6 (9.1)	40.3 (9.3)
**Gender**					
	Male	106 (49.3)	126 (54.1)	75 (64.1)	69 (67.6)
	Female	109 (50.7)	107 (45.9)	42 (35.9)	33 (32.4)
**Marital status**					
	Never married	91 (42.3)	80 (34.3)	46 (39.3)	30 (29.4)
	Married	115 (53.5)	146 (62.7)	67 (57.3)	67 (65.7)
	Divorced or bereaved	9 (4.2)	7 (3.0)	4 (3.4)	5 (4.9)
**Occupation**					
	Manager	42 (19.5)	45 (19.3)	35 (29.9)	39 (38.2)
	Professional	49 (22.8)	65 (27.9)	25 (21.4)	20 (19.6)
	Clerical	101 (47.0)	96 (41.2)	41 (35.0)	37 (36.3)
	Production	1 (0.5)	1 (0.4)	0 (0.0)	0 (0.0)
	Sales	18 (8.4)	20 (8.6)	12 (10.3)	5 (4.9)
	Others	4 (1.9)	6 (2.6)	4 (3.4)	1 (1.0)
**Education**					
	High school	9 (4.2)	13 (5.6)	5 (4.3)	5 (4.9)
	Some college	38 (17.7)	38 (16.3)	23 (19.7)	14 (13.7)
	University	151 (70.2)	165 (70.8)	72 (61.5)	65 (63.7)
	Graduate school	17 (7.9)	17 (7.3)	17 (14.5)	18 (17.6)
**Chronic disease**					
	Yes	19 (8.8)	25 (10.7)	19 (16.2)	16 (15.7)
	No	196 (91.2)	208 (89.3)	98 (83.8)	86 (84.3)

^a^0.1%-6% annual percentage yield.

^b^≥10% annual percentage yield.

### Effects of Internet-Based Computerized Cognitive Behavioral Therapy by Time Preference Subgroups

In the whole sample (n=667), the iCBT program showed a significant pooled intervention effect on BDI-II (*t*_548.79_=−3.36, *P*<.01) and K6 (*t*_551.08_=−2.70, *P*=.01) at 12-month follow-up. For each follow-up, iCBT showed a significant effect on BDI-II (*t*_1238.9_=−2.61, *P*=.01) at 3-months, on BDI-II (*t*_1232.1_=−3.18, *P*<.01) and K6 (*t*_1253.8_=−2.36, *P*=.02) at 6-months, and on BDI-II (*t*_862.64_=−3.19, *P*<.01) and K6 (*t*_875.92_=−2.37, *P*=.02) at 12-month follow-up.

In the lower time preference subgroup (n=448), iCBT program showed a significant pooled effect on BDI-II (*t*_374.06_=−3.31, *P*<.01) and K6 (*t*_368.30_=−3.09, *P*<.01) at 12-month follow-up. For each follow-up, iCBT showed a significant effect on BDI-II (*t*_842.44_=−2.25, *P*=.02) at 6-month and on BDI-II (*t*_583.45_=−3.32, *P*<.01) and K6 (*t*_652.08_=−2.81, *P*=.01) at 12-month follow-up. The other combinations were not statistically significant (data available upon request).

In the higher time preference subgroup (n=219), the pooled effects were not significant for both BDI-II (*t*_172.52_=−1.10, *P*=.27) and K6 (*t*_180.18_=−0.39, *P*=.70). For each follow-up, iCBT showed a significant effect on BDI-II (*t*_384.05_=−2.54, *P*=.01) and K6 (*t*_426.28_=−2.04, *P*=.04) only at 3-months and on BDI-II (*t*_385.61_=−2.44, *P*=.02) at 6-month follow-up. The other combinations were not statistically significant (data available upon request).

**Table 2:** Average scores of depressive symptoms (Beck Depression Inventory-II [BDI-II] and Kessler’s Psychological Distress Scale 6 [K6]) at baseline and 1-, 6-, and 12-month follow-up.

Subgroup and follow-up		Intervention							Control			
		n			K6, mean (SD)		BDI-II, mean (SD)			n	K6, mean (SD)	BDI-II, mean (SD)
**Lower time preference group^a^**												
	T1^b^		215			6.3 (4.5)		12.7 (8.6)		233	6.0 (4.3)	12.2 (8.7)
	T2^c^		143			5.9 (4.8)		10.5 (8.6)		202	6.0 (4.3)	11.7 (8.2)
	T3^d^		152			5.7 (4.6)		10.1 (8.2)		204	6.3 (5.0)	11.6 (9.0)
	T4^e^		135			5.6 (5.0)		10.6 (9.5)		187	6.7 (4.8)	12.7 (9.9)
**Higher time preference group^f^**												
	T1			117	5.9 (5.2)		12.2 (8.4)			102	6.5 (4.9)	13.7 (10.2)
	T2			78	4.8 (4.5)		9.5 (7.9)			86	6.7 (5.1)	13.9 (10.9)
	T3			79	4.9 (4.5)		9.4 (7.6)			93	6.9 (5.5)	13.9 (11.1)
	T4			75	5.9 (5.0)		10.8 (9.9)			86	6.3 (4.6)	12.5 (10.5)

^a^0.1%-6% annual percentage yield.

^b^T1: baseline.

^c^T2: 3-month follow-up.

^d^T3: 6-month follow-up.

^e^T4: 12-month follow-up.

^f^≥10% annual percentage yield.

**Table 3:** Estimated mean difference^a^, pooled SD^b^, and effect size^c^ between groups.

Subgroup and follow-up		K6^d^				BDI-II^e^		
		Estimated Δ^a^	Pooled SD	Effect size	Estimated Δ^a^		Pooled SD	Effect size
**Lower time preference group^f^**								
	T2^g^-T1^h^	−0.02	3.69	−0.01	−0.98		6.69	−0.15
	T3^i^-T1	−0.78	4.16	−0.19	−1.68		7.21	−0.23
	T4^j^-T1	−1.21	4.01	−0.30	−2.63		8.02	−0.33
**Higher time preference group^k^**								
	T2-T1	−1.13	3.77	−0.30	−2.55		6.85	−0.37
	T3-T1	−0.88	4.38	−0.20	−2.59		6.36	−0.41
	T4-T1	−0.17	4.40	−0.04	−0.91		7.40	−0.12

^a^Estimated means were calculated using a MIXED procedure.

^b^Pooled SDs were calculated using respondents those who completed the questionnaire at baseline and at follow-ups.

^c^Effect sizes were calculated by dividing estimated mean difference by pooled SD.

^d^K6: Kessler’s Psychological Distress Scale.

^e^BDI-II: Beck Depression Inventory-II.

^f^0.1%-6% annual percentage yield.

^g^T2, 3-month follow-up.

^h^T1, baseline.

^i^T3, 6-month follow-up.

^j^T4, 12-month follow-up.

^k^≥10% annual percentage yield.

**Table 4:** Three-way interaction effects of the internet-based cognitive behavioral therapy, time, and time preference on Beck Depression Inventory-II (BDI-II) and Kessler’s Psychological Distress Scale (K6).

Scale and follow-up		Crude					Gender, occupational status, and education adjusted				
		Effect	95% CI	SE	*t*	*P*	Effect	95% CI	SE	*t*	*P*
**K6**											
	3 months^a^	1.96	0.31 to 3.61	0.84	2.33	.02	2.12	0.46 to 3.77	0.84	2.51	.01
	6 months^a^	0.98	−0.67 to 2.63	0.84	1.16	.25	1.14	−0.51 to 2.79	0.84	1.35	.18
	12 months^a^	−0.19	−1.88 to 1.50	0.86	−0.22	.83	−0.01	−1.70 to 1.68	0.86	−0.01	.99
	Pooled^b^	−0.06	−0.45 to 0.34	0.20	−0.27	.79	−0.02	−0.42 to 0.38	0.20	−0.11	.91
**BDI-II**											
	3 months^a^	3.57	0.43 to 6.72	1.60	2.23	.03	3.75	0.60 to 6.91	1.61	2.33	.02
	6 months^a^	2.87	−0.28 to 6.01	1.60	1.79	.07	3.07	−0.08 to 6.22	1.61	1.91	.06
	12 months^a^	0.23	−2.97 to 3.44	1.64	0.14	.89	0.44	−2.78 to 3.65	1.64	0.27	.79
	Pooled^b^	0.01	−0.75 to 0.78	0.39	0.04	.97	0.05	−0.72 to 0.82	0.39	0.13	.90

^a^A mixed model for repeated measures analysis of variance model analyses was conducted to estimate a three-way interaction effect among intervention, time, and time preference.

^b^A mixed model for repeated measures conditional growth model analyses was conducted to estimate a three-way interaction effect.

**Table 5:** Progress of learning in the internet-based cognitive behavioral therapy program in the two subgroups.

Contents	Lower time preference (n=215), n (%)		Higher time preference (n=117), n (%)	
	Completers of lessons	Submitters of homework	Completers of lessons	Submitters of homework
Lesson (L)1: *Learning about stress*	186 (86.5)	124 (57.7)	103 (88.0)	70 (59.8)
L2: *Knack for self-case formulation based on* *cognitive behavioral model*	181 (84.2)	85 (39.5)	97 (82.9)	51 (43.6)
L3: *Try cognitive restructuring part 1*	167 (77.7)	84 (39.1)	87 (74.4)	48 (41.0)
L4: *Try cognitive restructuring part 2*	153 (71.2)	68 (31.6)	80 (68.4)	37 (31.6)
L5: *Knack for communication*	142 (66.0)	54 (25.1)	76 (65.0)	34 (29.1)
L6: *How to solve your problem effectively*	138 (64.2)	55 (25.6)	69 (59.0)	28 (23.9)
All 6 lessons	136 (63.3)	37 (17.2)	68 (58.1)	20 (17.1)

### Process Evaluation

Table 5 shows the process evaluation indicators of iCBT programs for the lower and higher time preference subgroups. Most participants in the intervention group completed Lesson 1 (186/215, 86.5% in the lower time preference group and 103/117, 88.0% in the higher time preference group), and about 60% in both subgroups (124/215 in the lower time preference group and 70/117 in the higher time preference group) submitted their homework after completing this lesson. The proportion of those who completed lessons and submitted homework gradually decreased during the later lessons. About 60% in both subgroups (136/215 in the lower time preference group and 68/117 in the higher time preference group) completed all 6 lessons, while only about 17% of them (37/215 in the lower time preference group and 20/117 in the higher time preference group) submitted all 6 homework assignments. In the lower time preference group, the average number of lessons that the respondents received was 4.5 and the average number of homework assignments submitted was 2.2. Of all participants, 77.7% (177/215) completed at least 3 lessons, and 38.1% (82/215) submitted at least 3 homework assignments. In the higher time preference group, the average number of lessons that the respondents received was 4.4 and the average number of homework assignments submitted was 2.3. In total, 75.2% (88/117) participants completed at least 3 lessons, and 40.2% (47/117) participants submitted at least 3 homework assignments. There were no differences of completers of lessons or submitters of homework of the iCBT program in both subgroups.

## Discussion

### Principal Findings

This RCT examined the effects of iCBT on improving nonclinical depressive symptoms at 3-, 6-, and 12-month follow-ups among healthy workers by lower and higher time preference subgroups in Japan. As a result, the three-way interaction effect of iCBT was significant for nonclinical depressive symptoms at 3-month follow-up, after adjusting for gender, occupational status, and education. In the higher time preference subgroup, iCBT showed a significant intervention effect on nonclinical depressive symptoms at 3- and 6-month follow-ups, while the pooled effect was not significant. On the other hand, in the lower time preference subgroup, iCBT showed significant ESs on nonclinical depressive symptoms at 6- and 12-month follow-ups. The iCBT program showed a significant pooled effect on nonclinical depressive symptoms at 12-month follow-up.

To our knowledge, this is the first RCT that has demonstrated the effect of an iCBT on improving nonclinical depressive symptoms, specifically targeting workers with lower or higher time preference. iCBT showed a significantly higher effect for improving nonclinical depressive symptoms in the higher time preference subgroup than the lower time preference subgroup at 3-month follow-up. Workers with higher time preference may more easily change their cognition or behavior, but these changes persisted for only a short period. The pooled effect of iCBT was significant only in the lower time preference subgroup. Workers with lower time preference may be more likely to keep their cognitive or behavioral changes for a longer period.

### Comparison with Prior Work

This study showed a difference in the intervention effect of iCBT between the higher time preference subgroup and the lower time preference subgroup. However, in the process evaluation, there were no differences between completers of lessons and submitters of homework of the iCBT program in both subgroups. Our findings caused us to reject the hypothesis that participants with higher time preference were less likely to follow the program.

Previous systematic reviews suggested that higher time preference was associated with poor responses to health promotion interventions such as dietary and weight loss programs [17,18]. However, this study showed that the higher time preference subgroup experienced a faster improvement in depressive symptoms than the lower time preference subgroup. Our study did not support the hypothesis that participants with higher time preferences were less likely to react to the program. Rather these participants exhibited significant mood improvements within a short (3-month) period. Workers with higher time preference may be more likely to change their behavior following engagement with an intervention that is immediately useful for treating their problems such as CBT, rather than an intervention that leads to long-term benefits such as healthy behaviors for preventing lifestyle-related diseases. Learning during the early period enhanced the intervention effects for the lower time preference subgroups.

The intervention effects of iCBT were less persistent among workers with higher long-term time preferences (eg, over 6 months). These findings support the hypothesis that the effect of CBT is not persistent among people with higher time preferences. Workers with higher time preferences may experience difficulty in maintaining their cognitive and behavioral changes. Workers with higher time preferences may stop using their new CBT-related perspectives or behaviors when their problems are solved (ie, improvement of nonclinical depressive symptoms). They may underestimate the future risk for a recurrence of the problems and not keep practicing a preventive effort. A follow-up program providing incentives (eg, allocating points or giving a prize as a reward) may reinforce continuing activities, making the iCBT program more effective even after 6 months for workers with higher time preferences.

These findings may contribute to further understanding of behavioral characteristics of people based on their (higher or lower) time preference. In this study, workers with higher time preferences were less likely to maintain the effects of a CBT-based program over the long-term, compared with those with lower time preference, while both groups engaged in learning to a similar extent. This pattern was consistent with previous reports on the impact of time preferences on health-related behaviors such as obesity and smoking [17-19]. This study expanded on already observed behavioral characteristics of individuals with higher time preferences, indicating that behavior patterns associated with higher time preference can be applied to the CBT-based programs. In addition, this study observed a very interesting pattern associated with higher time preference: the intervention effect was temporarily boosted among workers with higher time preferences, which was not seen for those with lower time preferences. This behavior pattern may be observed for other health-related behaviors such as diet, weight loss, and smoking cessation. Further research is warranted to examine the generalizability to other behaviors and the nature of this short-term boost effect. By utilizing the temporary boost of behavior changes to form sustainable changes it could be possible to develop an effective health promotion program especially targeting people with higher time preferences.

### Limitations

There are several limitations of this study that should be considered. First, we did not conduct a prestratification for randomization by time preference. The sample may be biased between the intervention and control groups in each subgroup. Second, participants were recruited from one corporate group in Japan. The participation rate was very low (835/20,000, 4.2%). Most participants were married, working in clerical positions, and university graduates. They had their own PCs or tablet computers in their offices or homes. The participants were also supposed to have experience using a PC and studying through Web-based programs. Higher education level may also help participants learn from the iCBT program. The generalization of these findings to the general working population is limited. Third, while we excluded those who had MDE before, the scores of depressive symptoms and psychological distress of the participants at baseline were relatively high. These findings may be more applicable to respondents with mild depression. Fourth, the dropout rates in this study were 27.9% (197/706), 25.2% (178/706), and 31.6% (223/706) at the 3-, 6-, and 12-month follow-ups, respectively. The dropout rates were higher in the intervention group than in the control group during the entire follow-up period. The dropouts may have caused a selection bias, particularly if the intervention group participants with higher levels of depression were more likely to quit the program. Fifth, it is possible that participants in the control group acquired information about the iCBT program from participants in the intervention group at the same workplace. This contamination could weaken the intervention effect. Sixth, all outcomes in this study were measured by self-report, which might have been affected by the perception of participants or by situational factors at work.

### Conclusions

The iCBT program was significantly better at improving nonclinical depressive symptoms in the higher time preference subgroup compared with the lower time preference subgroup at the 3-month follow-up. Workers with higher time preferences may easily change their cognition or behavior, but the change may persist for only a short period. On the other hand, the pooled effect of iCBT during the entire follow-up period was significant only in the lower time preference subgroup. Workers with lower time preferences may be likely to keep their cognitive or behavioral changes for a longer period. A further RCT with a precise design, such as stratified permuted-block randomization, should be conducted to test the potential different intervention effects of the iCBT program on nonclinical depressive symptoms between lower and higher time preference subgroups.

## Appendix

Multimedia Appendix 1 The time preference questionnaire used in the study and the number of respondents in each category.

Multimedia Appendix 2 CONSORT-EHEALTH checklist (V 1.6.1).

## Abbreviations

BDI-II: Beck Depression Inventory-II
CBT: cognitive behavioral therapy
ES: effect size
iCBT: internet-based computerized cognitive behavioral therapy
K6: Kessler’s Psychological Distress Scale
MDD: major depressive disorder
MDE: major depressive episode
RCT: randomized controlled trial
