# Supplementary material for: Differences in the Effect of Internet-Based Cognitive Behavioral Therapy for Improving Nonclinical Depressive Symptoms Among Workers by Time Preference: Randomized Controlled Trial
Source: J Med Internet Res. 2018 Aug 10;20(8):e10231. doi: 10.2196/10231 (PMC6109227; doi:10.2196/10231)
Supplement: Multimedia Appendix 1 [file jmir_v20i8e10231_app1.pdf]

Multimedia appendix 1 The time preference questionnaire used in the study and the number of respondents in each category. Question: “Suppose you have two mutually-exclusive options to receive some money. You may choose Option “A”, to receive 1 million JPY in a month; or Option “B”, to receive a different amount in 13 months. Compare the amounts and delay until its receipt in Option “A” with Option “B” and indicate which option you would prefer for each pair of all nine choice pairs.”

Option A (Receipt in a month)	Option B (Receipt in 13 months)	Interest rate (Annual)	No. of participants who first chose the option B [a)]	Classification of time preference [b)]
JPY 1 million	JPY 950,000	−5%	1/6	Excluded
JPY 1 million	JPY 1 million	0%	20/12	Excluded
JPY 1 million	JPY 1,001,000	0.10%	58/67	Low
JPY 1 million	JPY 1,005,000	0.50%	27/23	Low
JPY 1 million	JPY 1,010,000	1%	42/37	Low
JPY 1 million	JPY 1,020,000	2%	14/25	Low
JPY 1 million	JPY 1,060,000	6%	74/81	Low
JPY 1 million	JPY 1,100,000	10%	51/54	High
JPY 1 million	Over JPY 1,100,000	Over 10%	39/24	High
	Never chose the option B	Over 10%	27/24	High

a) The number of participants in the intervention/control groups.
b) Low- and high-time preference subgroups were classified by using the median of time preference score at baseline. Participants who selected irrational options (interest rate, -5% or 0%) were excluded because these categories are irrational.
